# mTORC1-Mediated Angiogenesis is Required for the Development of Rosacea

**DOI:** 10.3389/fcell.2021.751785

**Published:** 2021-12-21

**Authors:** Qinqin Peng, Ke Sha, Yingzi Liu, Mengting Chen, San Xu, Hongfu Xie, Zhili Deng, Ji Li

**Affiliations:** ^1^ Department of Dermatology, Xiangya Hospital, Central South University, Changsha, China; ^2^ Hunan Key Laboratary of Aging Biology, Xiangya Hospital, Central South University, Changsha, China; ^3^ National Clinical Research Center for Geriatric Disorders, Xiangya Hospital, Central South University, Changsha, China; ^4^ Key Laboratory of Molecular Radiation Oncology of Hunan Province, Changsha, China; ^5^ Key Laboratory of Organ Injury, Aging and Regenerative Medicine of Hunan Province, Central South University, Changsha, China

**Keywords:** angiogenesis, mTORC1, rosacea, Cabozantinib, rapamycin, endothelial cells

## Abstract

Although multiple evidences suggest that angiogenesis is associated with the pathophysiology of rosacea, its role is still in debate. Here, we showed that angiogenesis was enhanced in skin lesions of both rosacea patients and LL37-induced rosacea-like mice. Inhibition of angiogenesis alleviated LL37-induced rosacea-like features in mice. Mechanistically, we showed that mTORC1 was activated in the endothelial cells of the lesional skin from rosacea patients and LL37-induced rosacea-like mouse model. Inhibition of mTORC1 decreased angiogenesis and blocked the development of rosacea in mice. On the contrary, hyperactivation of mTORC1 increased angiogenesis and exacerbated rosacea-like phenotypes. Our *in vitro* results further demonstrated that inhibition of mTORC1 signaling significantly declined LL37-induced tube formation of human endothelial cells. Taken together, our findings revealed that mTORC1-mediated angiogenesis responding to LL37 might be essential for the development of rosacea and targeting angiogenesis might be a novel potential therapy.

## Introduction

Rosacea is a commonly chronic facial inflammatory disorder with a prevalence ranging from less than 1% to up to 22% (Tan and Berg, 2013; Two et al., 2015). Cutaneous features include flushing, erythema, telangiectasis, papule, pustule and ocular manifestations, mainly impacting the skin of the centrofacial and periocular regions (Culp and Scheinfeld, 2009). According to the National Rosacea Society Expert Committee in 2017, the updated classification of rosacea is based on phonotypes linked to clinical manifestations and provides criterion of diagnosis and severity assessment of the disease (Gallo et al., 2018). Rosacea may negatively influence the patients’ physiology and psychology, resulting in an overall reduced quality of life.

To date, the pathogenesis of rosacea remains largely unclear and is thougth to be associated with dysfunction in immune response, dysregulation of nervous and vascular system (Steinhoff et al., 2011; Moustafa et al., 2014).Toll-like receptor 2 (TLR2), as a component of innate immunity, is highly expressed in keratinocytes of rosacea patients (Yamasaki et al., 2011). TLR2 stimulates enhanced production of Kallikrein 5 (KLK5) and KLK5 further processes cathelicidin into its active form LL37 which is deemed to play an important role in the vasodilation and angiogenesis of rosacea (Yamasaki et al., 2007; Yamasaki et al., 2011; Steinhoff et al., 2013). Additionally, flushing and telangiectasis are results of vascular dilation, while erythema may be a reflection of abnormal vasculature, namely angiogenesis (Sibenge and Gawkrodger, 1992; Guzman-Sanchez et al., 2007). Although some groups proposed that angiogenesis played an important role in the pathophysiology of rosacea (Gomaa et al., 2007; Huggenberger and Detmar, 2011), recent reports emphasized the increased vasodilation rather than angiogenesis in rosacea skin specimens (Schwab et al., 2011). Vascular endothelial growth factor (VEGF) is a known key mediator of angiogenesis and vasopermeability (Neufeld et al., 1994). Previous studies showed that VEGF expression, angiogenesis and lymphangiogenesis increased in rosacea skin lesions (Gomaa et al., 2007; Smith et al., 2007), while another study indicated that dilated vessels were evident, with no or slight upregulation of key angiogenic genes and limited angiogenesis in papulopustular rosacea (Schwab et al., 2011). Altogether, whether cutaneous angiogenesis is involved in the pathogenesis of rosacea or a just reflection of inflammation is a matter of controversy and further research is warranted in this area.

The mechanistic target of rapamycin mTOR is an atypical serine/threonine protein kinase constituting two distinct complexes named mTOR complex 1 (mTORC1) and 2 (mTORC2) (Laplante and Sabatini, 2012). As a pivotal regulator of metabolism and nutrient, the major function of mTORC1 responds to environmental stimuli to regulate cellular metabolism, growth and survival (Laplante and Sabatini, 2012; Saxton and Sabatini, 2017; Ardestani et al., 2018). mTORC1 phosphorylates the downstream effectors S6K1 and 4E-BP1, thus promoting associated protein synthesis (Saxton and Sabatini, 2017). The heterodimer consisting of tuberous sclerosis protein complex 1 (TSC1) and TSC2 is a key negative regulator of mTORC1 (Saxton and Sabatini, 2017). Our recent study has demonstrated that mTORC1 signaling played an important role in the pathogenesis of rosacea through forming a positive feedback loop between mTORC1 and cathelicidin (Deng et al., 2021). In addition, mTORC1 signaling is involved in some other skin diseases, such as atopic dermatitis (Naeem et al., 2017) and psoriasis (Buerger, 2018). Emerging evidence also demonstrated the association between mTORC1 and angiogenesis in other non-skin conditions (Bruning et al., 2018; Ding et al., 2018).

Here, we validated cutaneous angiogenesis in the pathogenesis of rosacea and identified a novel molecular mechanism that in endothelial cells mTORC1 signaling mediated the angiogenesis responding to LL37. Our findings have provided evidence for cutaneous angiogenesis as a potential therapeutic target in rosacea.

## Materials and Methods

### Human Specimens

All patients diagnosed with rosacea and healthy individuals were recruited by the Department of Dermatology in Xiangya Hospital of Central South University. Patients who had received antibiotic or other treatments within 3 months were excluded. Skin biopsies were taken and written informed consent was obtained from patients and controls. The study procedure was approved by the institutional review board (IRB) of Xiangya Hospital in accordance with the Helsinki Guidelines.

### Animal Experiments

BALB/c mice (purchased from Slack Company), the previous reported TSC2 knockout (TSC2^+/−^) and wild-type (WT) mice namely TSC2^+/+^ mice on C57BL/6 background (Deng et al., 2021) were housed and bred in standard specific pathogen-free laboratory conditions. DNA was extracted from mice tails for genotyping. Mice at the age of 7,8 weeks with the approval of the Institutional Animal Care and Use Committee at Xiangya Hospital of Central South University were used for all the experiments.

According to the previously described LL37-induced rosacea-like mouse model, mice were injected subcutaneously into the dorsal skin with 40 ul of LL37 peptide (320 mM) with 12 h intervals and skin inflammation was assessed 12 h after the last injection (Yamasaki et al., 2007). Rapamycin was reconstituted in absolute ethanol diluted in aqueous solution of 5.2% Tween-80 (9005-65-6, Sigma) and 5.2% of Poly ethylene glycol (25322-68-3, Sigma) before injection. The final volume of all injections was 50 μl. 4 mg/kg Rapamycin (RAPA) was injected intraperitoneally to BALB/c mice once per day 1 day before the injection of LL37 until the termination of LL37 injection. And 30 mg/kg Cabozantinib (Cabo) was administered intraperitoneally into BALB/c mice once a day. Skin tissues were collected for subsequent experiments.

### Reagents

Cathelicidin LL37 peptide, the amino acid sequence of which is LLGDFFRKSKEKIGKEFKRIVQRIKDFLRNLVPRTES, was synthesized by Sangon Biological Technology (Shanghai, China) followed by purification to >95% purity by HPLC. Cabo and RAPA were purchased from Selleck Chemicals (Shanghai, China).

### Total RNA Extraction and Real Time Quantitative-PCR

Total RNA was extracted from human biopsies and mice tissues using TRIzol Reagent (Thermo Fisher Scientific) and purified RNA was quantified by a Nanodrop spectrophotometer. cDNA was synthesized with the Maxima H Minus First Strand cDNA Synthesis Kit (Thermo Fisher Scientific). Real time quantitative-PCR (RT-qPCR) was carried out using the CFX Connect Real-Time PCR Detection System (Bio-Rad, California, United States) with iTaq Universal SYBR Green Supermix (Bio-Rad, California, United States) under the following RT-qPCR cycling parameters: 95°C for 60 s, then 95°C for 15 s and 60°C for 60 s for 40 cycles, 65°C for 5 s. Relative gene expression was standardized to GAPDH and calculated by the 2^−ΔΔCT^ method. The following primers were used: 5′-TGT​TGC​CAT​CAA​TGA​CCC​CTT-3′ and 5′-CTC​CAC​GAC​GTA​CTC​AGC​G-3′ for human GAPDH; 5′-AGG​GCA​GAA​TCA​TCA​CGA​AGT-3′ and 5′-AGG​GTC​TCG​ATT​GGA​TGG​CA-3′ for human *VEGFA*; 5′-GAG​ATG​TCC​CTG​GAA​GAA​CAC​A-3′ and 5′-GAG​TGG​GAT​GGG​TGA​TGT​CAG-3′ for human *VEGFB*; 5′-AGG​TCG​GTG​TGA​ACG​GAT​TTG-3′ and 5′-TGT​AGA​CCA​TGT​AGT​TGA​GGT​CA-3′ for mouse GAPDH; 5′- TAT​TCA​GCG​GAC​TCA​CCA​GC-3′ and 5′- AAC​CAA​CCT​CCT​CAA​ACC​GT-3′ for mouse *Vegf*.

### Histology and Immunohistochemistry

Human and mouse skin tissues were fixed in formalin and embedded in paraffin. 5 um sections were subject to hematoxylin and eosin staining or CD31 immunohistochemical staining as previously described (Wu et al., 2018; Zhao et al., 2018). Mouse anti CD31 antibody (MA5-13188, Invitrogen) was used and the dilution ratio was 1:100. Slides counterstained with H&E and immunochemical staining were imaged by an Eclipse Ni-U Upright Microscope (Nikon, Japan) under a magnification of 10 × 10.The number of inflammatory or CD31-positive vessels was counted in six randomly selected high power fields (HPFs).

### Immunofluorescence

The immunofluorescence staining was performed as previously described (Chen et al., 2019). The human and mouse skin samples were embedded in OCT (Tissue Tek) and sectioned to 8–10 um. Frozen sections were fixed in 4% paraformaldehyde, blocked with 5% normal donkey serum, permeabilized in 0.2% Triton X-100 and then incubated with primary antibodies. DAPI was counterstained to visualize the nuclei. Antibodies used and dilution ratio were rat anti CD31 (558736, BD Biosciences, 1:100) and rabbit anti pS6 (5364, CST, 1:200 or 1:800). Images were acquired by an Eclipse Ni-U Upright Microscope (Nikon, Japan) under a magnification of 10 × 20 or 10 × 40. The number of CD31^+^ or pS6^+^ vessels was counted in six randomly selected HPFs.

### Cell Culture and Treatment

HUVECs (Human Umbilical Vein Endothelial Cells, ATCC) were cultured in endothelial cell basal medium (EBM2, PromoCell) containing 2% fetal bovine serum supplemented with endothelial cell growth medium supplement (ECGM-2, PromoCell) and 1% (v/v) penicillin/streptomycin (Gibco) at 37°C in a humidified CO_2_ incubator. Different doses of LL37, ranging from 0 to 8 μM, were added to the medium with VEGF-treated group as a positive control. RAPA was administrated 2h before the LL37 stimulation.

### Tube Formation Assay

The effect of LL37 ± RAPA on angiogenesis was evaluated by tube formation assay as previously described (Michaloski et al., 2016). 96-well plates were coated with matrigel (BD Biosciences, United States) per well at 37°C for 1 h and then seeded with 2 × 10^4^ HUVECs in the presence of VEGF (20 ng/ml) or not. EBM2 culture medium with different doses of LL37 (0–8 μM) ± RAPA (50 nM) was added to the plates and VEGF-treated group was regarded as a positive control. After 12-hour incubation, the tube structure was observed and imaged with a microscope. Quantitative analysis of the tube formation was assessed by counting the number of tubes in six randomly selected fields and images were taken with an Eclipse Ts2 Inverted Routine Microscope (Nikon, Japan) under a magnification of 10 × 4 using ImageJ software version 1.51j8.

### Western Blot

HUVECs (2 × 10^5^ cells) initially seeded in 6-well plates were lysed in RIPA buffer (Thermo scientific Scientific) containing protease inhibitor cocktail (Thermo scientific Scientific) after the stimulation of LL37 and RAPA. Protein extracts were quantified and subjected to 10% SDS-PAGE and transferred to polyvinylidene fluoride (PVDF) membranes (Millipore, Bedford, MA, United States). The membrane was blocked with 5% skimmed milk and then incubated with primary antibodies pS6 (5364, CST, 1:1000) and S6 (2217, CST, 1:1000) overnight with Tubulin (sc-5286, Santa Cruz Biotechnology, 1:1000) taken as a loading control. The membrane was incubated with horseradish peroxidase (HRP)-conjugated second antibodies for 1h and protein was visualized with ECL Western Blotting detection kit (Millipore) using Image Lab software (Bio-Rad). Quantification of band intensity was analyzed by ImageJ software.

### Gene Set Enrichment Analysis

GenePattern 2.0 was utilized to process RNA-seq data for gene set enrichment analysis, which was run using the default settings (100 iterations, weighted). Gene enrichment was conducted on RNA-seq datasets of human skin biopsies from healthy individuals (*n* = 10) and rosacea patients (*n* = 10), described in our previous study (Deng et al., 2021). The gene set associated with angiogenesis signalling pathway downloaded from The Molecular Signatures Database (MSigDB) was tested. A false discovery rate *p* value <0.05 was considered significant. Sequencing data of rosacea pati ents have been deposited in the genome sequence archive under accession number HRA000378. Sequencing data for rosacea like mouse model have been deposited at the GEO database, under accession number GSE147950.

### Statistical Analysis

Data is presented as the mean ± standard error (SEM). Data were analyzed by two-tailed unpaired Student’s *t*-test between two groups or by One-way ANOVA when comparing more than two groups. GraphPad Prism (Graph Pad, San Diego, United States) was used to analyze all data. *p* < 0.05 was considered statistically significant. ns, not significant; *p < 0.05; **p < 0.01; ***p < 0.001.

## Results

### Angiogenesis Is Increased in the Lesional Skin of Rosacea

**FIGURE 1:**
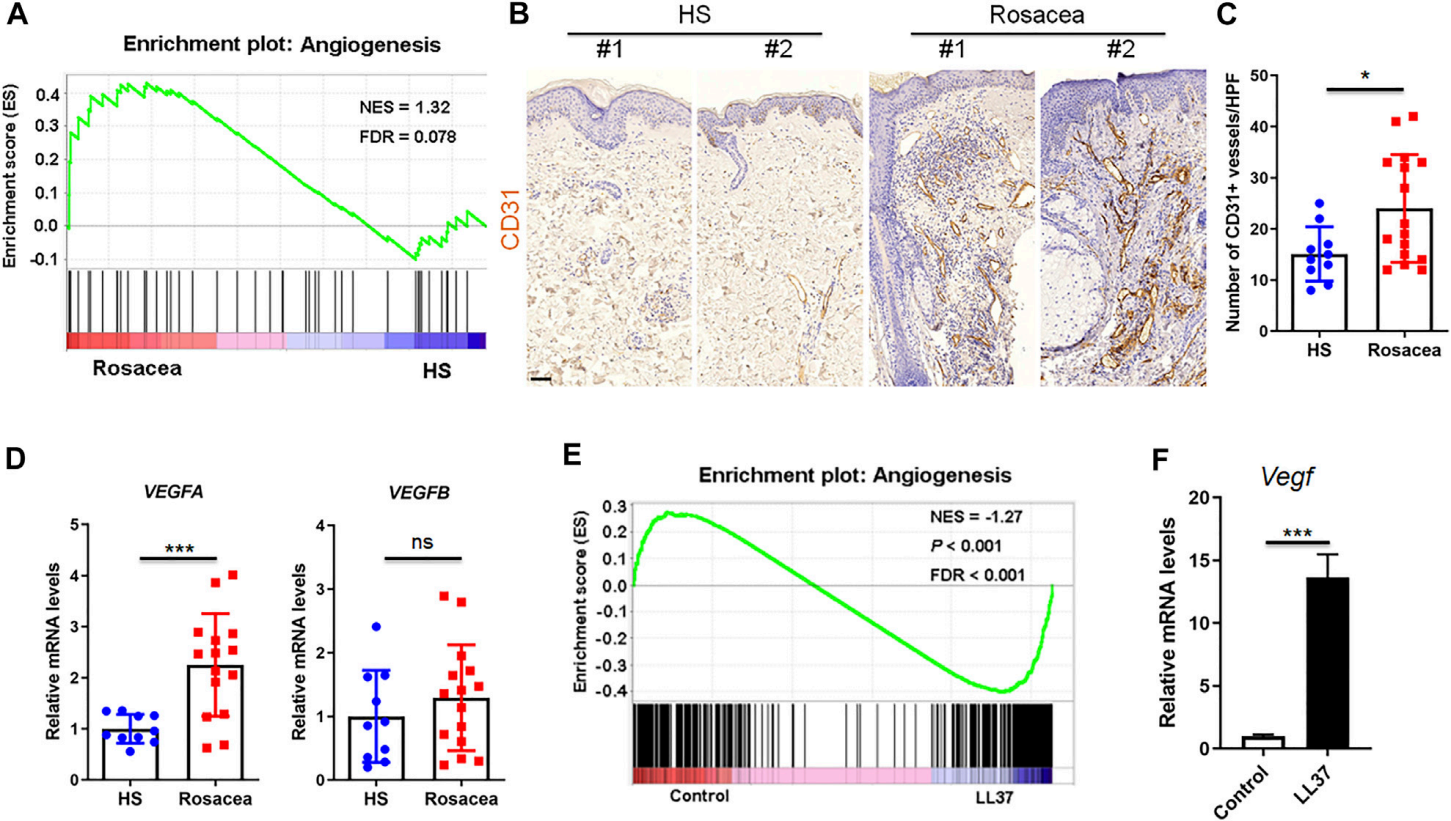
Angiogenesis is upregulated in rosacea skin samples. **(A)** GSEA plots of angiogenesis from RNA-Seq data in rosacea lesions and healthy skin (HS). The plots are displayed with normalized enrichment score (NES) and false discovery rate (FDR). **(B,C)** Immunohistochemical staining of CD31 and quantitative analysis of CD31-positive vessels on skin sections from healthy skin and rosacea lesions. Scale bar: 50 μ0. **(D)** mRNA expression of *VEGFA* and *VEGFB* in skin tissues from healthy skin and rosacea lesions detected by RT–qPCR. **(E)** GSEA plots of angiogenesis from RNA-Seq data in skin tissues from LL37-induced rosacea mouse model and control mouse. **(F)** *Vegf* mRNA expression in skin samples from LL37-induced rosacea-like mouse model and control mouse by RT-qPCR. Data represent mean ± SEM from three independent experiments. Statistical significance was determined by two-tailed unpaired Student’s *t*-test **(C,D,F)**. NS, not significant. *p < 0.05 and ***p < 0.001.

The role of angiogenesis in rosacea is controversial (Steinhoff et al., 2013). To clarify the vascularization in rosacea, we performed Gene set enrichment analysis between rosacea and healthy skin biopsies on our previously uploaded RNA sequencing data in the genome sequence archive (Deng et al., 2021) and identified enrichment of genes related to angiogenesis in lesional skin from rosacea patients compared with healthy individuals (Figure 1A). Consistently, immunohistochemistry of CD31, a marker for vascular endothelium, also revealed increased number of CD31-positive vessels in the lesional skin of rosacea patients (Figures 1B,C). As VEGF is known as a potent angiogenic factor, we also detected the mRNA levels of *VEGFA* and *VEGFB* by RT-qPCR. *VEGFA*, rather than *VEGFB* was highly expressed in rosacea lesions (Figure 1D).

Meanwhile, through interrogating our previous transcriptional data acquired from the GEO database (Deng et al., 2021), we discovered angiogenic genes were significantly enriched in skin samples from LL37-induced rosacea-like mouse model compared with control mouse via GSEA (Figure 1E). The *Vegf* mRNA level in LL37-induced skin samples was dramatically higher than controls (Figure 1F). Thus, we conclude that increased angiogenesis presents in rosacea.

### Inhibition of Angiogenesis Alleviates the Rosacea-like Phenotypes Induced by LL37 in Mice

**FIGURE 2:**
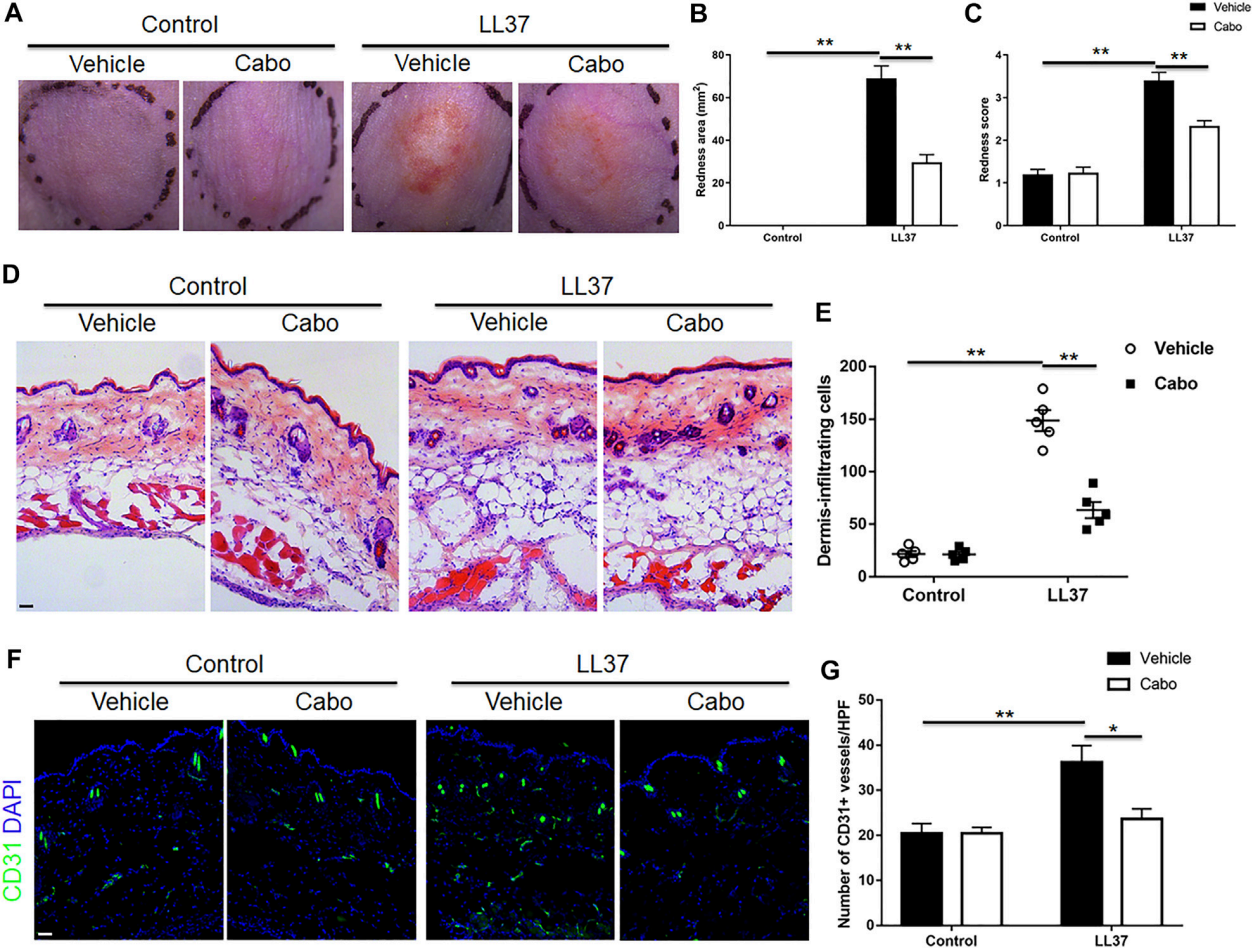
Inhibition of angiogenesis by Cabo alleviates LL37-induced rosacea-like phenotype. **(A)** Representative photographs of dorsal skin of mice from different groups. **(B,C)** Quantitative analysis of the redness area **(B)** and score of erythema **(C)** in dorsal skin of mice. **(D,E)** H&E staining and quantitative analysis of the number of cell infiltration. Scale bar: 50 μm. **(F,G)** Immunofluorescence staining with CD31 and quantitation of CD31^+^ vessels in skin tissues from four groups. Scale bar: 50 μm. Data represent mean ± SEM from three independent experiments. Statistical significance was determined by One-way ANOVA **(B,C,E,G)**. *p < 0.05 and **p < 0.01.

To detect the role of angiogenesis in the pathogenesis of rosacea, a previously described experimental LL37-induced rosacea-like mouse model was utilized (Yamasaki et al., 2007), and Cabo, a VEGFR2 inhibitor was used to inhibit the cutaneous angiogenesis in mice. As expected, LL37-induced rosacea-like lesions in mouse skin confirmed the successful establishment of the mouse model. Our results also showed that Cabo administration grossly ameliorated the rosacea-like skin phenotype in mice (Figure 2A). The quantification of redness area and score according to the previously described calculation method (Kim et al., 2015) showed obvious reduction in both Cabo and LL37-treated group comparing to solely LL37-treated control (Figures 2B,C). Upon closer histological analysis, we observed a significant inflammatory cell infiltration in LL37-induced mouse skin as previously reported (Deng et al., 2021), which was notably blocked by Cabo treatment (Figures 2D,E). Additionally, immuostaining result showed CD31^+^ endothelial cells increased prominently upon LL37 induction and Cabo application resulted in large decrease in its number, further validating the inhibitory effect of Cabo on LL37-induced cutaneous angiogenesis in mouse skin (Figures 2F,G). These results indicate angiogenesis is an important pathogenic mechanism in promoting the development of rosacea.

### mTORC1 Mediates Angiogenesis in the Development of Rosacea

**FIGURE 3:**
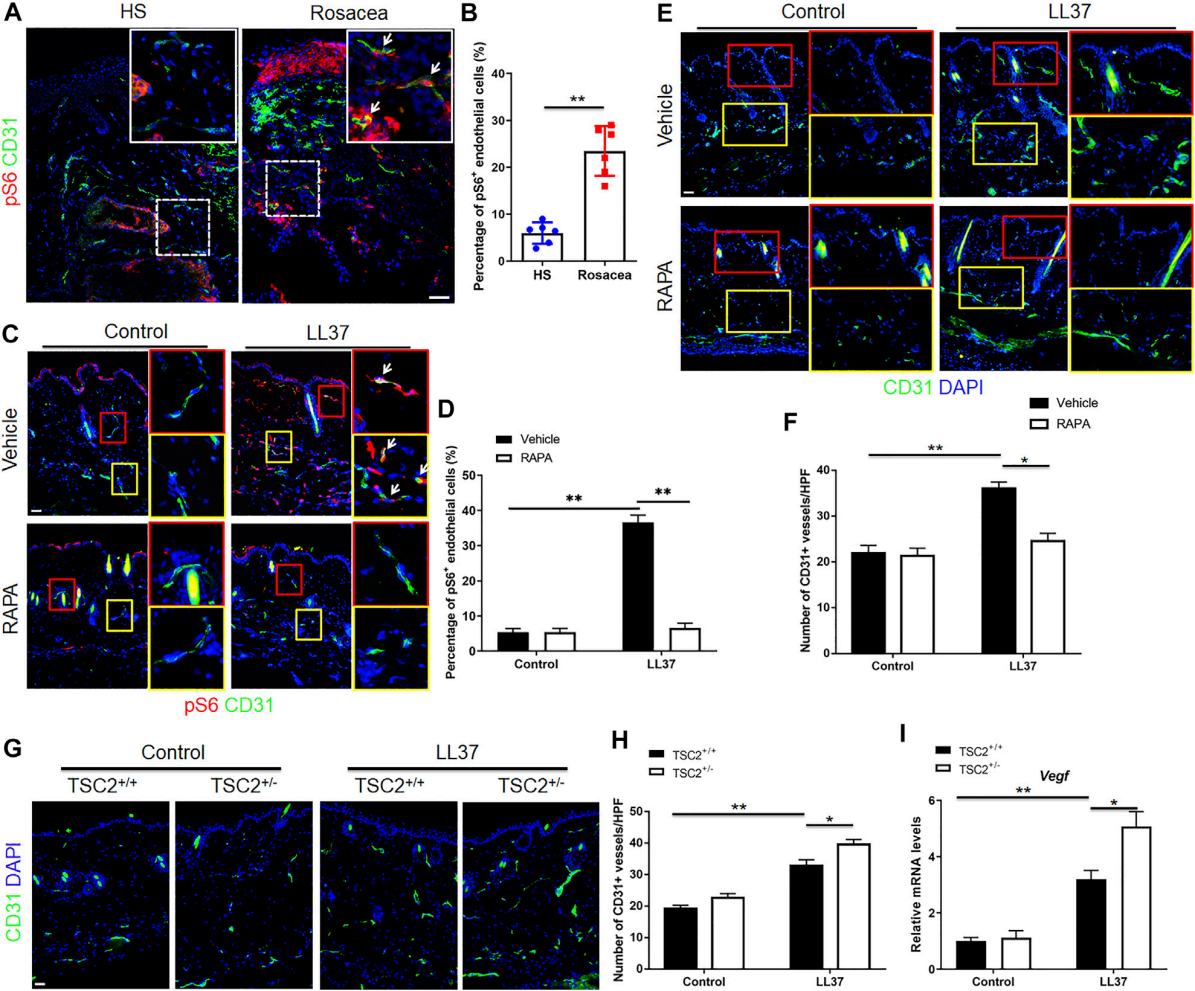
mTORC1 signaling regulates LL37-induced cutaneous angiogenesis *in vivo*. **(A,B)** Immunostaining of pS6 and CD31 **(A)**, and quantitative analysis of pS6 and CD31 double positive cells (indicated by white arrows) **(B)** in skin tissues from healthy skin and rosacea patients. **(C,D)** Immunostaining of pS6 and CD31, and quantitation of pS6 and CD31 double positive cells (indicated by white arrows) in mice skin tissues treated with LL37 and/or RAPA. **(E,F)** Immunostaining of CD31, and quantitative analysis of the number of CD31^+^ vessels in control and LL37-treated rosacea-like mouse model treated with or without RAPA. **(G,H)** Immunostaining of CD31, and quantitation of CD31^+^ vessels in WT and TSC2^+/−^ mouse skin treated with or without LL37. **(I)** *Vegf* mRNA expression in WT and TSC2^+/−^ mouse skin treated with or without LL37 by RT-qPCR. **(A,C,E)** Right panels indicate the magnified picture in the left box with the same color. Scale bar: 50 μm. Data represent mean ± SEM from three independent experiments. Two-tailed unpaired Student’s t-test **(B)** or 1-way ANOVA with Bonferroni’s post hoc test **(D,F,H,I)** was used *p < 0.05 and **p < 0.01.

As shown in our previous study, mTORC1 signaling was hyperactivated in the lesional skin of human rosacea patients, including endothelial cells (shown in pS6 immunohistochemistry result) (Deng et al., 2021). And inhibition of mTORC1 signaling blocked rosacea development, while rosacea-like phenotype aggravated in LL37-treated TSC2^+/−^ mice (Deng et al., 2021). To determine whether activation of mTORC1 signaling was associated with angiogenesis in rosacea, we first detected the activity of mTORC1 in endothelial cells in the lesional skin of rosacea patients and mouse model via co-immunostaining of pS6 and CD31. pS6 is a classical marker of mTORC1 activation (Linke et al., 2017). Increased pS6 expression in CD31 positive cells was observed in the dermis, indicating mTORC1 activation in CD31^+^ endothelial cells in both human rosacea and rosacea-like mouse model skin lesions (Figures 3A–D). We then asked if mTORC1 signaling plays a role in the angiogenesis of rosacea. So we inhibited the expression of mTORC1 with RAPA and evaluated its effect on angiogenesis in LL37-induced rosacea-like mouse model. The mTORC1 inhibitory effect of RAPA was validated by immunoblot analysis (Supplementary Figure S1A). As expected, RAPA treatment remarkably inhibited the mTORC1 activation and decreased the number of CD31^+^ vessels in skin tissues of LL37-induced mice compared to vehicle treatment (Figures 3C–F). TSC2 is a key negative regulator of mTORC1 (Ardestani et al., 2018), and mTORC1 is hyperactivated in the skin of TSC2^+/−^ mice as previously described (Deng et al., 2021). Accordingly, we detected the activation of mTORC1 by immunostaing of pS6 in skin samples from TSC2^+/−^ and TSC2^+/+^ control mice and confirmed the result (Supplementary Figure S1B). We found an increase in CD31^+^ vessels in TSC2^+/−^ mice induced by LL37 comparing to that in TSC2^+/+^ control mice (Figures 3G,H). The mRNA expression of *Vegf* responding to LL37 in TSC2^+/−^ mice also increased (Figure 3I). We further tested whether Cabo could influence mTORC1 signaling in rosacea-like mouse model and discovered that there was no difference between these two groups treated with Cabo or not (Supplementary Figure S1C). Thus, these findings indicate that mTORC1 signaling is essential in regulating cutaneous angiogenesis in the pathogenesis of rosacea.

### LL37 Induces Angiogenesis via mTORC1 Signaling in Human Endothelial Cells

**FIGURE 4:**
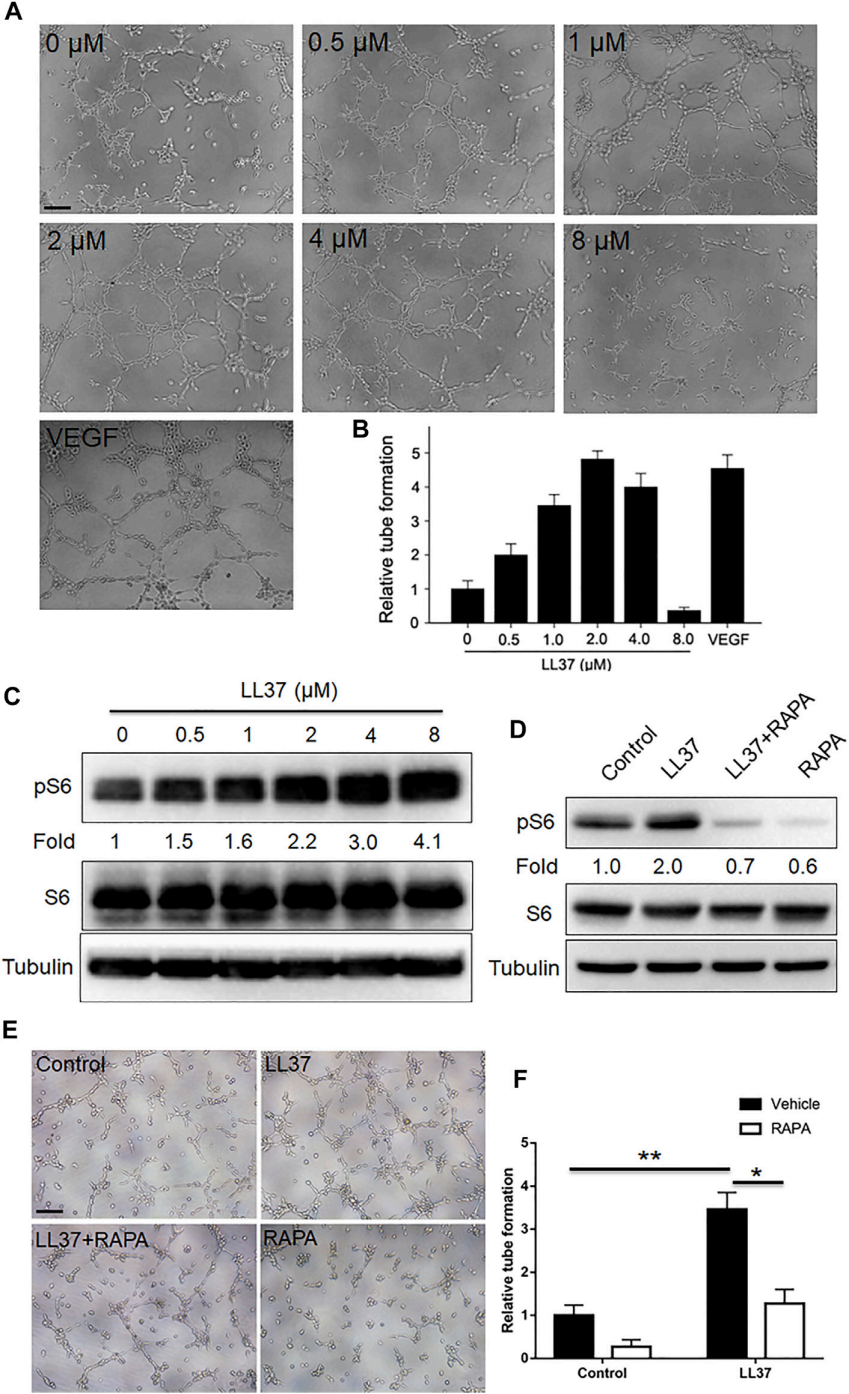
mTORC1 signaling regulates the tube formation mediated by LL37 in HUVECs. **(A)** HUVECs tube formation assay in presence of LL37. VEGF-treated group was set as a positive control. **(B)** Quantitative analysis of the tubular structure in **(A)**. **(C)** pS6 and S6 protein levels in LL37-treated HUVECs by western blotting. Tubulin was taken as a loading control. pS6 protein level was analyzed relative to total S6. **(D)** pS6 and S6 protein levels in LL37 and/or RAPA-treated HUVECs. Tubulin was taken as a loading control. pS6 protein level was analyzed relative to total S6. **(E)** HUVECs tube formation assay treated with LL37 and/or RAPA. **(F)** Quantitative analysis of the tubular structure in **(E)**. Scale bar: 50 μm. Data represent mean ± SEM from three independent experiments. Statistical significance was determined by One-way ANOVA **(B,F)**. *p < 0.05 and **p < 0.01.

Mounting evidence showed that mTORC1 signaling played an important role in vascular function and formation (He et al., 2020; Reho et al., 2021). Since mTORC1 signaling regulates cutaneous angiogenesis in LL37-induced rosacea-like mice, *in vitro* tube formation assay was employed to further examine this effect. Our results showed that low concentration of LL37 induced tube formation of HUVECs *in vitro* with its tubular formation induction capacity peaking at 2 uM concentration, while 8 uM LL37 inhibited tube formation (Figures 4A,B). And LL37 induced mTORC1 activation in HUVECs in a concentration-dependent manner (Figure 4C). Thus, we selected 2 uM LL37 for subsequent experiments. To test whether mTORC1 is required for the tube formation of HUVECs induced by LL37 *in vitro*, we treated HUVECs with RAPA in LL37-induced tube formation model. Our data showed that inhibition of mTORC1 markedly reduced the tube formation of HUVECs induced by LL37 (Figures 4D–F). These results establish that LL37 induces angiogenesis through activation of mTORC1 signaling.

## Discussion

Although dysfunction of vascular system is associated with the pathophysiology of rosacea, the role of angiogenesis in rosacea is still controversial and its detailed molecular mechanism has not been reported yet. Here, we confirmed the increased angiogenesis by combined transcriptome and immunohistochemistry analysis in skin lesions from rosacea patients and LL37-induced rosacea-like mouse model. We further discovered angiogenesis was a promoting factor in the development of rosacea. Our findings eventually highlighted the importance of angiogenesis in the pathogenesis of rosacea and revealed mTORC1 signaling as the downstream effector that mediated LL37-induced angiogenesis.

We first validated that upregulated angiogenesis was evident in rosacea skin lesions. Increased blood vessels laid a pathological foundation for flushing and erythema in accordance with previous proclamation which suggested that angiogenesis was related to the pathophysiology of rosacea (Aroni et al., 2008; Steinhoff et al., 2013). Related studies have also reported increased VEGFA levels as well as enhanced angiogenesis in lesional skin of rosacea (Gomaa et al., 2007; Smith et al., 2007). However, another report revealed that vasodilation was evident in all subtypes, while angiogenic key genes were rarely modulated and angiogenesis was found only in papulopustular rosacea (Schwab et al., 2011). Our study showed that vasoactive molecule VEGFA mRNA expression was enhanced in rosacea patients. These differences might be attributed to the selection bias of skin samples from rosacea patients and healthy individuals or sample size.

As relevant studies on the effect of angiogenesis inhibition on rosacea development have not been published, we used a previously described rosacea-like mouse model via intradermal injection of LL37 and discovered VEGFR2 inhibitor Cabo treatment significantly reduced the severity of erythema, immune cell infiltration and angiogenesis in the dorsal skin of mice induced by LL37. This result provided further evidence for involvement of angiogenesis in the pathogenesis of rosacea.

Our previous study showed that mTORC1 signaling hyperactivated mainly in the epidermis of rosacea (Deng et al., 2021). In the present study, we identified that mTORC1 activity increased in endothelial cells of rosacea patients and mouse model. Despite that mTORC1 signaling was reported to take a role in the pathogenesis of rosacea and other autoimmune dermatoses (Naeem et al., 2017; Buerger, 2018), the correlation between mTORC1 and angiogenesis in rosacea has not been elucidated. We co-stained skin sections from human and mouse samples with pS6 and CD31 antibodies and found pS6 was increased in endothelial cells of rosacea patients and LL37-induced mice. This implied a correlation between mTORC1 and angiogenesis in rosacea.

To further investigate whether mTORC1 signaling regulates angiogenesis in rosacea, we treated LL37-induced rosacea model with intraperitoneal injection of RAPA, a well-known mTORC1 inhibitor. Inhibition of mTORC1 resulted in notable alleviation of LL37-induced vasculature. Vice versa, hyperactivating mTORC1 signaling via TSC2 knockout as previously described (Deng et al., 2021) enhanced the number of CD31-positive vascular endothelial cells induced by LL37.These results suggested mTORC1 acted as an regulator of LL37-induced angiogenesis in rosacea.

Furthermore, previous studies proved mTORC1 signaling upregulated the angiogenesis in other non-autoimmune dermatoses, especially in tumors (Espona-Fiedler et al., 2012; Wang et al., 2020). In addition, mTOR inhibition by RAPA decreased microvessel sprout formation in human dermal microvascular endothelial cells and microvessel density in mice bearing soft tissue sarcoma cell line xenograft tumors, producing synergistic effects with radiation (Murphy et al., 2009). It also documented that LL37 induced angiogenesis via PGE2-EP3 signaling (Murphy et al., 2009). Yet, the role of mTORC1 signaling in LL37-induced angiogenesis remains unclear. In agreement with the previous studies, LL37 induced tube formation of HUVECs *in vitro*. And RAPA treatment grossly reduced the number of tubes induced by LL37, indicating mTORC1 regulated LL37-induced angiogenic response *in vitro*. Considering our previous study demonstrated that mTORC1 promotes cathelicidin LL37 production in keratinocytes via a positive feedback loop, which leads to accumulation of LL37 (Deng et al., 2021), we speculate that excess LL37 from keratinocytes induces angiogenesis via activation of mTORC1 signaling in endothelial cells, eventually aggravating the development of rosacea.

In summary, our work revealed that angiogenesis played an important role in the pathogenesis of rosacea and identified mTORC1 signaling as an mediator of LL37-induced angiogenesis in rosacea, thus establishing a LL37-mTORC1-angiogenesis axis in the pathogenesis of rosacea. Our findings provided fundamental evidence for targeting angiogenesis in clinical therapy of rosacea. Further investigation into the role of angiogenesis in rosacea and the precise molecular mechanism by which mTORC1 regulates angiogenesis is required.

## Data Availability

Publicly available datasets were analyzed in this study. This data can be found here: GSA database and accession number HRA000378 (http://bigd.big.ac.cn/gsa-human/browse/HRA000378), GEO database and accession number GSE147950 (https://www.ncbi.nlm.nih.gov/geo/).
