# Supplementary material for: mTORC1-Mediated Angiogenesis is Required for the Development of Rosacea
Source: Front Cell Dev Biol. 2021 Dec 21;9:751785. doi: 10.3389/fcell.2021.751785 (PMC8724421; doi:10.3389/fcell.2021.751785)
Supplement: Supplementary file 1 [file DataSheet1.PDF]

Supplementary figure and figure legends

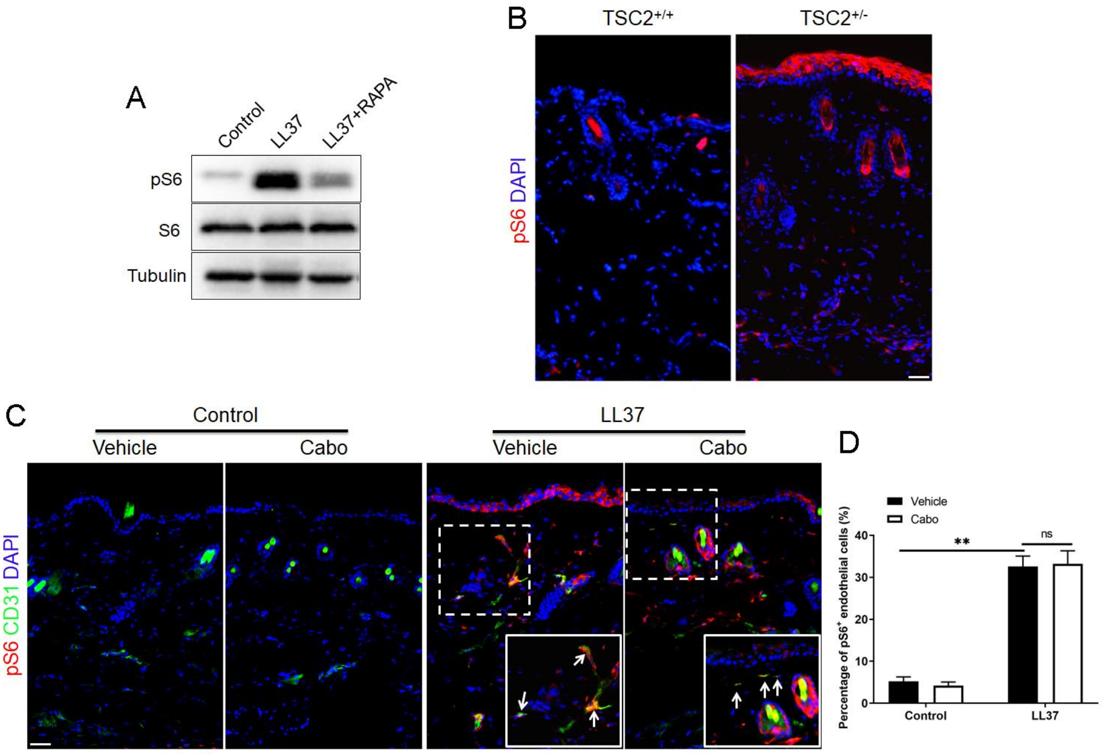

Supplementary Figure 1. mTORC1 mediates angiogenesis in the development of rosacea.

(A) Immunoblot analysis of pS6 and S6 in mouse skin treated with or without LL37. Tubulin was taken as a loading control. (B) Immunofluorescence staining for pS6 in skin samples from WT and $TSC2^{+/-}$ mouse. (C, D) Immunofluorescence staining for pS6 and CD31 and quantitative analysis of pS6 and CD31 double positive cells in control and LL37-treated rosacea-like mouse model treated with or without Cabo. Scale bar: 50 μm. Statistical significance was determined by One-way ANOVA (D). $^{**}p < 0.01$.
